# Accuracy of a New Platelet Count System (PLT-F) Depends on the Staining Property of Its Reagents

**DOI:** 10.1371/journal.pone.0141311

**Published:** 2015-10-23

**Authors:** Atsushi Wada, Yuri Takagi, Mari Kono, Takashi Morikawa

**Affiliations:** Scientific Research Division, Scientific Affairs, Sysmex Corporation, 1-3-2 Murotani, Nishi-ku, Kobe, Hyogo 651–2241, Japan; Centro Cardiologico Monzino IRCCS, ITALY

## Abstract

**Background:**

Platelet count is essential for the diagnosis and management of hemostasis abnormalities. Although existing platelet count methods installed in common hematology analyzers can correctly count platelets in normal blood samples, they tend to miscount platelets in some abnormal samples. The newly developed PLT-F channel in the XN-Series hematology analyzer (Sysmex) has been reported to be a reliable platelet count system, even in abnormal samples. However, how the PLT-F platelet counting system achieves such accuracy has not been described in scientific articles.

**Methods:**

Isolated platelets, erythrocytes, and fragmented erythrocytes were examined using an automated hematology analyzer. The samples were labeled by combining PLT-F reagents and anti-CD62p, CD63, Grp75, Calreticulin, CD41, or CD61 antibody, and analyzed using confocal laser scanning microscopy or flow cytometry.

**Results:**

The PLT-F system correctly discriminated platelets in erythrocytes. Its reagents strongly stained some intraplatelet organelles labeled with anti-Grp75, but only faintly stained the plasma membrane of both platelets and erythrocytes. Microscopic observation and flow cytometric examination revealed that all of these strongly stained cells were also labeled with platelet-specific anti-CD41 and anti-CD61 antibodies.

**Conclusions:**

This study revealed that the staining property of the PLT-F reagents, by which platelets and fragmented erythrocytes are clearly distinguished, contributes to the platelet-counting accuracy of the PLT-F system.

## Introduction

Platelet count, which is usually performed on whole blood using automated hematology analyzers in clinical laboratories, is essential for the diagnosis and management of hemostasis abnormalities [1–5]. The first-generation automated platelet counters, which were introduced in clinical laboratories nearly half a century ago, were developed to replace the labor-intensive and irreproducible routine manual platelet counting [6]. Some improved first-generation systems, such as PLT-I (Sysmex, Kobe, Japan), CDS-I (Abbott Laboratories, North Chicago, IL, USA), and LH-750 impedance (Beckman Coulter, Brea, CA, USA), are installed in modern automated hematology analyzers and are still used in clinical laboratories. However, the first-generation systems cannot distinguish platelets from other particles of the same size because these systems depend on only particle size, which is measured using electric impedance.

To resolve this issue, a precise platelet count method using platelet-specific monoclonal antibodies and multiparameter flow cytometry was developed [7, 8]. However, because of its costly operation and slightly complicated procedures, the immunological method has not become a standard method in general clinical laboratories. Nonetheless, this accurate method has developed into the reference method of platelet counting.

Second-generation automated platelet count systems, based on flow cytometry, were first developed over 10 years ago. These systems, such as PLT-O (Sysmex), CDS-O (Abbott), and ADVIA optical counting method (Siemens), are installed in common automated hematology analyzers currently used in clinical laboratories. These methods can count platelets correctly in the majority of blood samples, some exceptions are unusual samples with low platelet concentration or those containing abnormal cells, such as megathrombocytes [9–16]. In particular, blood specimens from severely burned patients are among the most difficult samples for common platelet counters because they contain too many fragmented erythrocytes (RBC ghosts), which are the same size as platelets. Although the most reliable platelet counting method for such specimens is flow cytometry with platelet-specific antibodies, this method remains difficult to use routinely in clinical laboratories. For these reasons, a fast, reliable, affordable, and easy-to-use platelet count system is required.

The next-generation automatic platelet count method, PLT-F, which is based on fluorescent labeling and flow cytometry, has been installed in the newly developed XN-Series hematology analyzer (Sysmex). The PLT-F system is superior to the two existing routine methods: the second-generation PLT-O and the first-generation PLT-I. This method is highly correlated with the immunological reference method, even for samples with low platelet concentrations [17–20]. In addition, the PLT-F system can enumerate platelets in blood samples from severe burn injury patients with the same accuracy as the reference method [19]. However, how the system achieves such accuracy has not been described in a scientific manner.

In this study, we compared two scattergrams, one of the new PLT-F system and the other of the existing PLT-O system, obtained using the same whole-blood specimen. Then, we compared the staining property of new PLT-F reagents to that of existing PLT-O reagents. Finally, we examined whether the PLT-F system can discriminate platelets from fragmented erythrocytes accurately, in comparison with the immunological method.

## Materials and Methods

### Preparation of platelets, erythrocytes, and fragmented erythrocytes

The whole-blood samples from five volunteers were drawn into blood collection tubes with K2-EDTA (Terumo, Tokyo, Japan). Pure platelet-rich plasma (PRP) was prepared by centrifugation as described previously [21]. After collecting PRP, erythrocytes were collected from the rest of the blood sample using the Lymphocyte Separation Solution d = 1.119 (Nacalai Tesque, Kyoto, Japan). Then, the cells were washed with phosphate-buffered saline (PBS). Washed erythrocytes were then heated at 50°C for 15 min to prepare fragmented erythrocytes. These samples were examined with the automated hematology analyzer XN-2000 (Sysmex) and XE-2100 (Sysmex). These samples were stained with May-Grünwald-Giemsa staining solution (Muto Pure Chemicals, Tokyo, Japan) and photographed using a BX-50 microscope with a DP25 microscope camera (Olympus, Tokyo, Japan).

### Statistical analysis of the difference in platelet counts between PLT-F, PLT-I, and PLT-O

Whole-blood specimens were collected from two donors. The PRP and erythrocyte fraction were prepared from each blood specimen as described above. Control or fragmented erythrocyte (fRBC) fraction, PRP, and PBS were mixed in the proportion of 1:1:8 as a control RBC-PRP or fRBC-PRP mixture. Then each mixture was examined 10 times in a row with the XN-2000 using its three platelet-counting methods, PLT-F, PLT-I, and PLT-O. The means of platelet numbers among the three methods (PLT-F, PLT-I, and PLT-O) were compared with a one-way analysis of variance (ANOVA) (Pairwise comparisons using t-tests with pooled SD; P value adjustment method: Bonferroni). Then, the means of the control and fRBC mixtures, measured with the same counting method, were compared using a two-sample t-test. A p-value equal to or below 0.05 was considered to be statistically significant. All statistical analyses were performed with EZR (Saitama Medical Center, Jichi Medical University, Saitama, Japan, version 1.30) [22], which is a graphical user interface for R (The R Foundation for Statistical Computing, Vienna, Austria, version 3.2.2; http://www.R-project.org/). More precisely, EZR is a modified version of R commander (version 2.2–0) designed to add statistical functions frequently used in biostatistics.

### Confocal laser scanning microscopy

Samples were labeled with FITC anti-human CD41 monoclonal antibody P2 (Beckman-Coulter IM0649U) or FITC anti-human CD61 monoclonal antibody SZ21 (Beckman-Coulter IM1758) at a final concentration of 20 μg/ml in 1% bovine serum albumin (SIGMA)/PBS (BSA/PBS), and were placed on a poly-L-lysine (Sigma-Aldrich, St. Louis, MO, USA)-coated glass-bottomed dish (Matsunami Glass, Osaka, Japan) and incubated for 30 min at room temperature (RT). For staining with either the PLT-F or PLT-O reagents, the samples on the PLL-coated glass were covered with either the PLT-F or PLT-O reagent mixture (PLT-F, CELLPACK DFL:Fluorocell PLT = 50:1; PLT-O, RET SEARCH Dilution buffer:RET SEARCH (II) staining dye = 50:1, Sysmex) for approximately 1 min at RT. Then, the samples were observed with a confocal laser scanning microscope system (IX81, Olympus; CSU-X1, Yokogawa Electric, Tokyo, Japan; ImagEM, Hamamatsu Photonics, Hamamatsu, Japan).

To analyze the intraplatelet staining property of the PLT-F reagent, we first placed 1/10 diluted PRP with PBS on Poly-L-Lysine coated glass-bottomed dish (D11131H, Matsunami) and allowed platelets to attach for more than 30 min at RT. After removal of the supernatant, the platelets on the glass were covered with 4% paraformaldehyde in PBS and allowed to fix for 20 min at RT. After removal of the fixation solution, platelets were rinsed in three changes of 100 mM glycine/PBS and permeabilized by 0.2% TritonX-100/PBS for 2 min. After the permeabilization solution was removed, the platelets were rinsed twice with PBS and covered with blocking solution (1% Bovine Serum Albumin (BSA) /PBS) for 30 min. Then, the blocking buffer was replaced with the primary antibody solution; both 1/20 anti-CD63-FITC (mouse monoclonal IgG1 clone CLBthromb/6, Beckman-Coulter) and 1/200 anti-Grp75 (rabbit monoclonal IgG1 clone D13H4, Cell Signaling Technology, Danvers, MA, USA), or both 1/20 anti-CD62p-FITC (mouse monoclonal IgG1 clone CLBGran/12, Beckman-Coulter) and anti-Calreticulin (rabbit monoclonal IgG clone D3E6, Cell Signaling Technology). Subsequently, he samples were incubated overnight at 4°C. After rinsing three times in PBS, platelets were incubated in a secondary antibody solution including both 1/400 Alexa Fluor 488 conjugated goat anti-mouse IgG (H+L) secondary antibody (A-11029, Thermo Fisher scientific, Waltham, MA, USA) and 1/400 Alexa Fluor 546 conjugated goat anti-rabbit IgG (H+L) secondary antibody (A-11035, Thermo Fisher scientific) in 1% BSA/PBS for 30 min at RT. After rinsing three times in PBS, platelets were covered with 2% Fluorocell-PLT (Sysmex) in PBS and observed with a confocal laser scanning microscopy FV-10i (Olympus).

### Flow cytometry

Equal volumes of platelets and fragmented erythrocytes were mixed. For both, 3 μl of FITC-labeled antibody (either anti-CD41 monoclonal antibody P2 (Beckman-Coulter IM0649U) or control IgG (Beckman-Coulter A07795)) and 3 μl of PE-labeled antibody (either anti-CD61 monoclonal antibody SZ21 (Beckman-Coulter IM3605) or control IgG (Beckman-Coulter A07796)) were added to 24 μl of the mixture and incubated for 30 min at RT. After labeling with both antibodies, the mixture was stained with 300 μl of the PLT-F reagent mixture for 90 sec at RT and analyzed using an EC800 cell analyzer (Sony, Tokyo, Japan).

### Ethics statement

This study was approved by Sysmex Ethics Committee. All participants provided their written informed consent to participate in this study according to the study protocol.

## Results

### Direct comparison of PLT-F and PLT-O scattergrams

**Fig 1:**
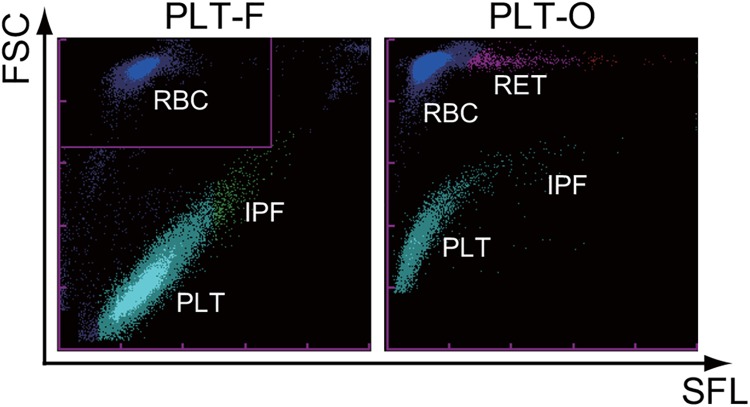
Typical scattergrams of the two flow cytometric platelet count channels on the XN-Series automated hematology analyzer. The same whole-blood sample from a healthy volunteer was measured at the same time on the XN-2000 automated hematology analyzer. Each panel shows PLT-F (left) and PLT-O (right) scattergrams. FSC: forward scattered light; SFL: side fluorescent; RBC: erythrocytes; PLT: platelets; IPF: immature platelet fraction; RET: reticulocytes.

First, we directly compared the two flow cytometric platelet count scattergrams (PLT-F and PLT-O), which were taken with the same XN-2000 analyzer at the same time from the same whole-blood sample from a healthy volunteer (Fig 1). Platelets and erythrocytes were clearly discriminated by the intensity of the forward-scattered light (vertical axis in Fig 1) on these two scattergrams. However, erythrocytes were stained differently by each fluorescent dye. Erythrocytes, which are displayed as a compact cluster on the PLT-F scattergram, formed a long-tailed cluster on the PLT-O scattergram. This comparison revealed that each dye from these two systems had different staining properties.

### Different staining properties of PLT-F and PLT-O reagents

**Fig 2:**
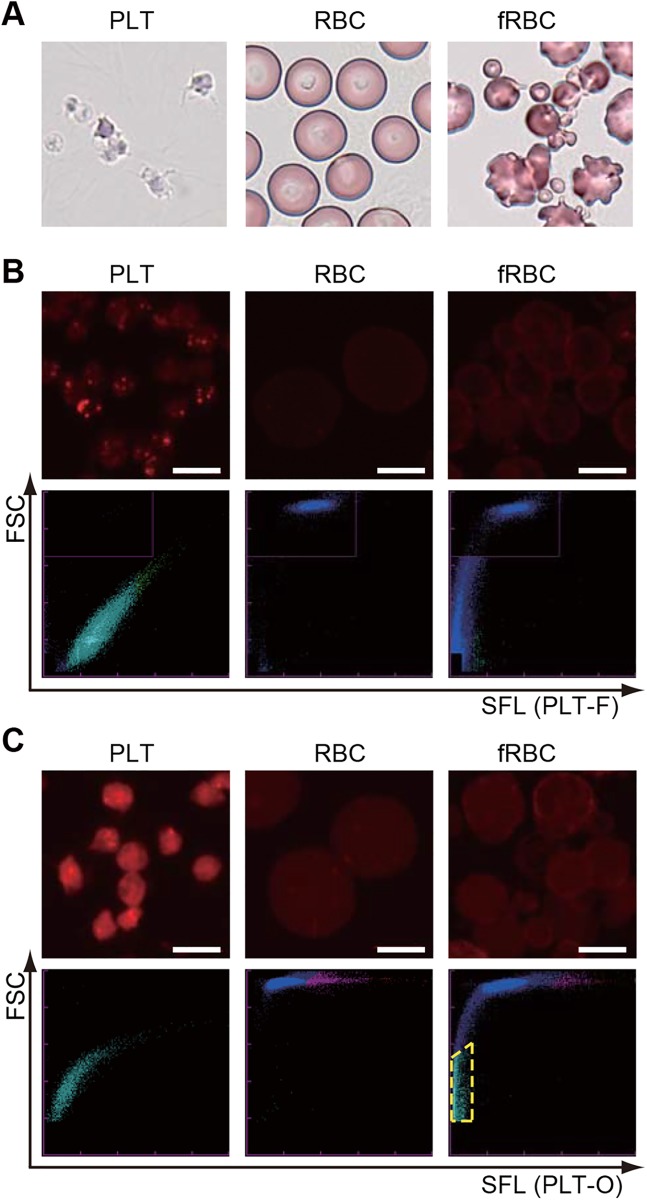
Platelets and fragmented erythrocytes were distinguished with PLT-F reagents. In each row, the left, middle, and right columns show platelets (PLT), erythrocytes (RBC), and fragmented erythrocytes (fRBC), respectively. (A) May-Giemsa-stained cells. (B) (Upper) PLT-F-stained cells. (Lower) PLT-F scattergrams. (C) (Upper) PLT-O-stained cells. (Lower) PLT-O scattergrams. Pale blue dots surrounded by yellow dashed line indicate fRBC misidentified as platelets. Bars: 5 μm.

Next, we examined the differences of staining properties between PLT-F and PLT-O reagents. For this purpose, we prepared platelet-rich plasma as isolated platelets, purified erythrocytes by density-gradient centrifugation, and fragmented erythrocytes (also known as RBC ghosts) by applying heat shock to purified erythrocytes (Fig 2A). Then, we stained each cell with either PLT-F or PLT-O reagents in the same manner as in the automated hematology analyzers, and observed them with a confocal laser scanning microscope (Fig 2B and 2C, upper panels). When we stained platelets with the PLT-F reagents, intraplatelet structures were strongly labeled (Fig 2B, upper left), but plasma membranes of both platelets and erythrocytes (and also fragmented erythrocytes) were only slightly stained (Fig 2B, upper panels). In contrast, PLT-O reagents stained not only platelets but also the plasma membrane of erythrocytes effectively (Fi 2C, upper). Both isolated platelets and erythrocytes were correctly identified in each scattergram (Fig 2B and 2C, lower, left and middle). Almost all of the fragmented erythrocytes were also recognized as RBC on the PLT-F scattergram, but a subset of them was misidentified as platelets on the PLT-O scattergram (Fig 2B and 2C, lower right, pale blue dots surrounded by yellow dashed line).

### Platelet counts of PLT-F were less affected by fragmented erythrocytes than PLT-O

**Fig 3:**
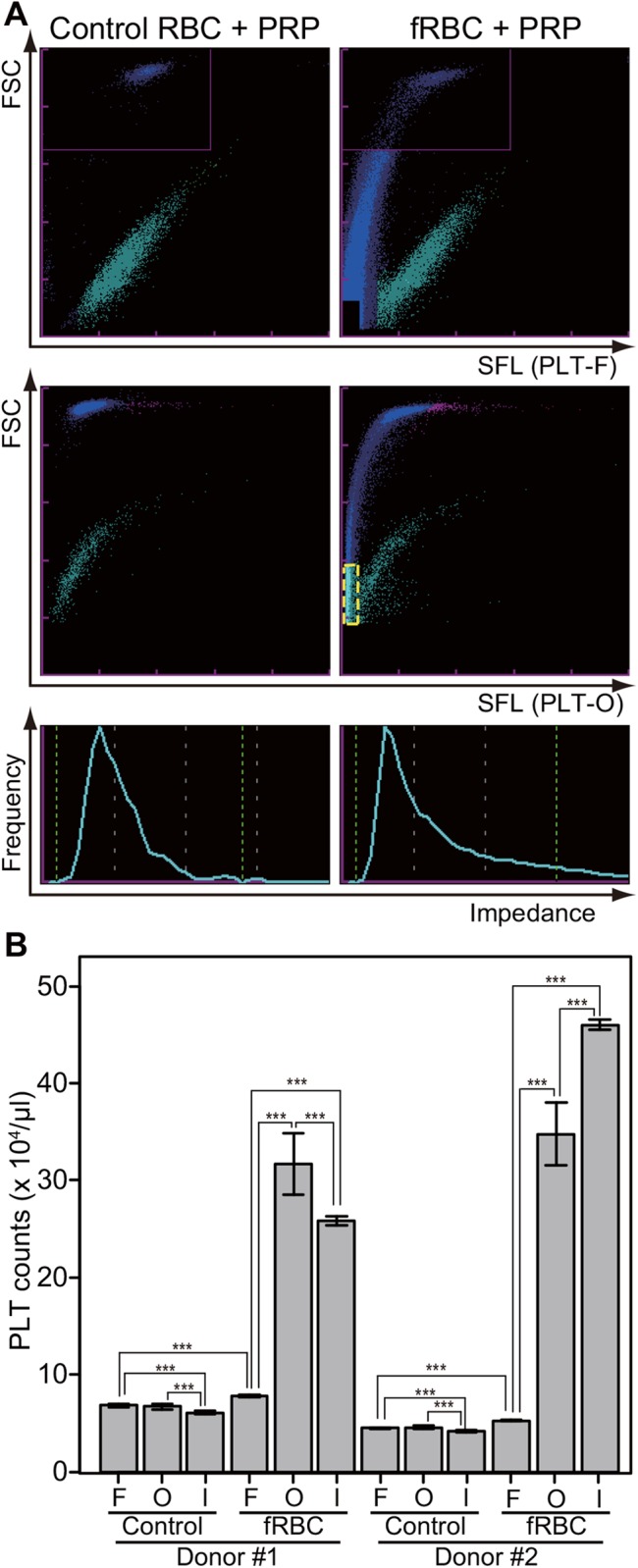
Comparison among three platelet counting methods using a fragmented RBC-PRP mixture. Three platelet counting methods (PLT-F, PLT-O, and PLT-I) were compared using a control or fragmented RBC (fRBC) and platelet rich plasma (PRP) mixture. The mixtures were made from two donors’ blood and examined ten times per sample. (A) Typical scattergrams from the three methods. Top: PLT-F, middle: PLT-O, bottom: PLT-I. Left: control RBC and PRP mixture, right: fRBC and PRP mixture. The pale blue dots surrounded by yellow dashed line indicate fRBCs misidentified as platelets. (B) Platelet count of the mixtures. Data are expressed as mean ± standard deviation of the mean. F: PLT-F, O: PLT-O, I: PLT-I. Control: control RBC and PRP mixture, fRBC: fragmented RBC and PRP mixture. Significant code *** means p < 0.001.

To confirm whether the PLT-F system could discriminate platelets from fragmented erythrocytes, we prepared and measured two types of artificial erythrocytes-platelets mixtures, which contained equal concentrations of platelets. The difference between the mixtures was that one included a control erythrocyte fraction composed of normal RBCs, and the other included a heat-treated erythrocyte fraction composed of mainly fragmented erythrocytes (fRBCs). Then, those mixtures were measured with three platelet counting methods, PLT-F, PLT-O, and PLT-I for ten times per sample. As shown in the scattergrams (Fig 3), PLT-F seemed to correctly distinguish fRBCs and platelets (Fig 3A, top-right), although the PLT-O scattergram misidentified small fragmented erythrocytes as platelets (Fig 3A, middle-right, pale blue dots surrounded by yellow dashed line). The PLT-I method also seemed to misidentify the fragmented erythrocytes as platelets (Fig 3A, bottom). When we statistically analyzed the mixture containing the control RBCs, no significant difference was found between the PLT-F and PLT-O methods (Fig 3B, Table 1, and Table A in S1 File). Although platelet counts with the PLT-F system significantly increased by 15% (Donor [23]) or 17% (Donor [24]) in the fRBC mixture, the rates of increase were much less than those of PLT-O (372%, 661%) or PLT-I (326%, 996%) (Fig 3B, Table 1, and Tables in S1 File).

**Table 1:** Mean, SD, and CV of platelet counts with PLT-F, PLT-O, and PLT-I.

	Donor [23]						Donor [24]					
	Control			fRBC			Control			fRBC		
	Mean	SD	CV	Mean	SD	CV	Mean	SD	CV	Mean	SD	CV
PLTF	6.82	0.15	2.27	7.84	0.13	1.61	4.51	0.06	1.26	5.27	0.09	1.80
PLTO	6.71	0.28	4.12	31.68	3.19	10.06	4.57	0.18	3.87	34.76	3.24	9.31
PLTI	6.06	0.19	3.13	25.84	0.44	1.70	4.20	0.11	2.51	46.02	0.56	1.22

SD: standard deviation, CV: coefficient of variation (%)

### Intraplatelet staining property of the PLT-F dye

**Fig 4:**
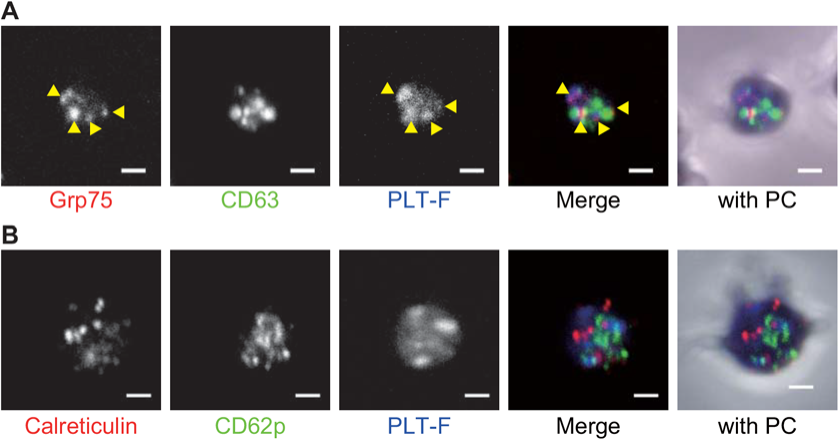
PLT-F stain signals were overlapped with anti-Grp75 staining signals. (A) Anti-Grp75 (red in merge channel), anti-CD63 (green in merge channel) and PLT-F (Blue in merge) triple staining. Yellow arrowhead indicates overlapped signals of anti-Grp75 and the PLT-F stain. (B) anti-calreticulin (Red in merge), CD62p (green in merge) and PLT-F signals (Blue in merge). (A and B) “with PC” channels shows merged images of immunofluorescence, PLT-F, and phase contrast images. PC stands for phase contrast. Bar = 1 μm.

As shown above, the PLT-F reagents strongly stained a few granules in every platelet (Fig 2B). To identify which granules the PLT-F stained, we performed immunofluorescence with different types of granule marker antibodies (anti-CD62p/P-selectin as an α-granule marker, anti-CD63 as a dense granule marker [25], anti-Grp75/mortalin as a possible marker for mitochondria [26], and anti-Calreticulin as a possible marker for residual endoplasmic reticulum and dense tubular system [27]). This was followed by PLT-F staining, which demonstrate that the anti-Grp75 antibody signals were overlapped with the PLT-F stained granules (Fig 4A, yellow arrowheads). In contrast, there was little evidence of a co-localization of PLT-F and the other three antibody signals (Fig 4A and 4B).

### Staining consistencies between PLT-F reagents and platelet-specific antibodies

**Fig 5:**
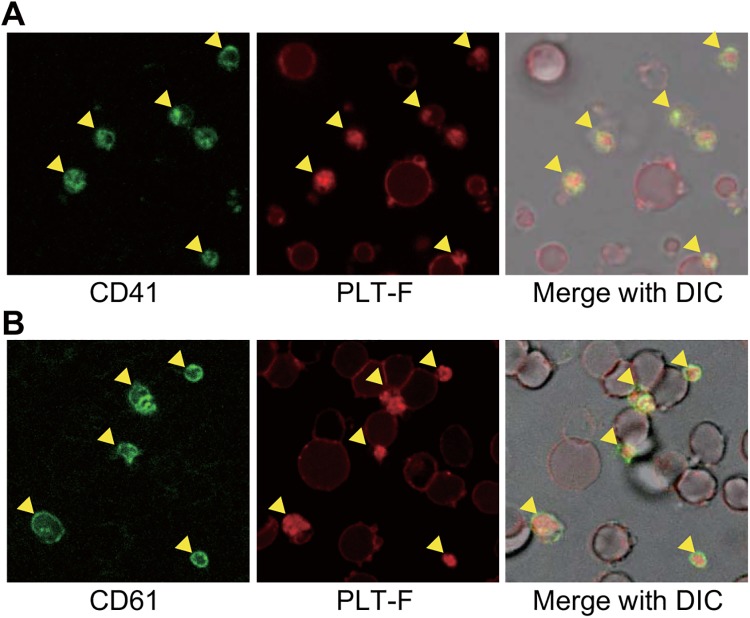
PLT-F reagents strongly stain CD41/CD61-positive platelets. In each row, a mixture of platelets and fragmented erythrocytes was stained with both platelet-specific antibodies (anti-CD41 (A) or anti-CD61 (B), left) and PLT-F reagents (middle). Yellow arrowheads indicate platelets.

To compare the staining specificity of PLT-F reagents with that of reference monoclonal antibodies, anti-CD41 and anti-CD61, we stained a platelet and fragmented erythrocyte mixture with both PLT-F reagents and each of the antibodies, and observed the staining patterns using confocal laser scanning microscopy (Fig 5). The PLT-F reagents strongly stained some small cells (Fig 5, middle, arrowheads), which completely overlapped with antibody-labeled platelets (Fig 5, left and right, arrowheads). However, the reagents faintly stained the plasma membrane of fragmented erythrocytes (Figs 2, 4 and 5). Thus, we concluded that PLT-F reagents, similar to the reference antibodies, could be used to distinguish platelets.

### Comparison of PLT-F reagents with platelet-specific antibodies using a multipurpose flow cytometer

Finally, we analyzed the platelet and fragmented erythrocyte mixture that was stained with FITC-anti-CD41, PE-anti-CD61, and the PLT-F reagents using a multipurpose flow cytometer. Through the analysis of the triple-stained sample, platelets (CD41^+^/CD61^+^ small particles) were distinguished from fragmented erythrocytes (CD41^-^/CD61^-^ particles) by the PLT-F staining (Fig 4A). Labeling by a single antibody (anti-CD41 or anti-CD61) and PLT-F reagents showed essentially the same results (Fig 4B and 4C). We also confirmed that platelets were not separated from fragmented erythrocytes on FSC/SSC scattergram or by the electrical impedance method (S1 Fig).

## Discussion

Through the direct comparison of two scattergrams from two platelet count systems, the new PLT-F system and the existing PLT-O system installed in XN-Series automated hematology analyzers (Fig 1), we noticed that each dye of these two systems had different staining properties for erythrocytes. In particular, reticulocytes, which are distinguished on the PLT-O scattergram as they show higher fluorescence than mature erythrocytes (pink and red dots in lower panels in Fig 2C), were not clearly distributed on the PLT-F scattergram (lower panels in Fig 2B). We previously demonstrated that the dyes used in the PLT-O method stained the intracellular components of the reticulocytes [28] as well as platelets (Fig 2C), whereas the PLT-F reagent would not stain the reticulocytes effectively, as indicated from the scattergrams in Fig 2B and 2C. Meanwhile, due to the same cationic properties of both dyes, because they were designed to stain nucleic acids, those dyes can stain anionic plasma membranes to a greater or lesser extent (Fig 2).

We examined the differences between these two reagents using isolated platelets, erythrocytes, and fragmented erythrocytes, which are the major confusing factors for platelet counting because of their similar size to platelets [9]. Indeed, a subset of the fragmented erythrocytes occupied the same region as platelets and was misidentified as platelets by the PLT-O system (Figs 2C and 3A). This misidentification may have been due to the staining property of the PLT-O reagents, which strongly stain the plasma membrane of erythrocytes (Fig 2C). The PLT-F reagents also stained some intraplatelet organelles, but the reagents only faintly stained erythrocyte membranes (Figs 2B and 4). Such differences are assumed to be due to the differences in the chemical properties of the dyes; the dyes in the PLT-F reagents have a phenoxazine backbone (Patent US20120315667 A1), whereas the dyes included in the PLT-O reagents were fluorescent polymethine (Patent US5891731 A). Such different properties of each dye could have caused the differences between the patterns of the PLT-F and PLT-O scattergrams (Figs 1–3), and the staining property of the PLT-F reagents would be the reason for the fine discrimination on the PLT-F scattergram between the platelets and fragmented erythrocytes (Figs 2B and 3). Quantitative analysis using the platelets-fragmented erythrocytes mixture confirmed the high discrimination performance of PLT-F system (Fig 3B, Table 1, and Tables in S1 File). When we measured the artificial blood containing both platelets and high concentrations of fragmented erythrocytes, the PLT-F system showed only a 15–17% increase in the platelet count, whereas the PLT-O and PLT-I systems show more than 300% increases (Fig 3B, Table 1 and Tables in S1 File). In addition, we found an unexpectedly high coefficient of variation (CV) value of platelet counts with the PLT-O method when we measured the platelet-fragmented erythrocyte mixture, whereas those of PLT-F were stable (Fig 3B and Table 1). Although we have not attempted to clarify the reason for the high CV value of the PLT-O system, we suggest that it may be due to the platelet-erythrocyte discrimination algorithm used in the system. Thus, we can conclude that the PLT-F is the most accurate platelet counting method among the three methods.

The PLT-F reagents strongly stained some types of granules in the platelets (Fig 2B and 2C). Because of the cationic properties of the dyes, we hypothesized that the granules might be platelet dense granules, since their insides are known to be acidic, or mitochondria for their own genomic DNA and various RNAs. Another suitable candidate would be a subset of α-granules. Detailed observation of the co-stained platelets revealed that signals from anti-Grp75, a mitochondrial marker protein, were overlapped with that of the PLT-F stain (Fig 3A). To our surprise, few significant overlap was found between the PLT-F stain and the other three granule marker antibody signals (anti-CD63 for dense granules, anti-CD62p for α-granules, or anti-Calreticulin for other α-granules, dense tubular system or other ER-derived organelles) (Fig 3). These results strongly suggest that the PLT-F dye mainly stains mitochondria and cytosolic messenger RNA in platelets. This conclusion is consistent with the fact that mature erythrocytes, and hence fragmented erythrocytes from them, do not have mitochondria, cytosolic or nuclear RNA/DNA, or any other organelle.

As shown above, the PLT-F reagents could be used as a tool to distinguish platelets from fragmented erythrocytes (Fig 2). In addition, some groups have reported that there was a good correlation between the PLT-F system and the immunological reference method using platelet-specific monoclonal antibodies 634 [18, 19] 758. These results suggest that the PLT-F reagents specifically stain platelets in a crowd of fragmented erythrocytes as well as the platelet-specific antibodies do. To test this possibility, we compared PLT-F staining with the signals by platelet-specific antibody, anti-CD41 or anti-CD61, using fluorescent microscopy. Because CD41 and CD61, also known as glycoproteins GPIIb and GPIIIa, are expressed on platelet membrane 669, the antibodies against these proteins are used in the reference method to count platelets [7, 8]. Although the PLT-F reagents stained erythrocytes faintly (Figs 2B and 5), the reagents strongly stained the platelets labeled by these antibodies (Fig 5). Previous reports confirm that PLT-F and immunological detection with CD antibodies had a strong relationship in blood specimens from clinical patients [17–20].

**Fig 6:**
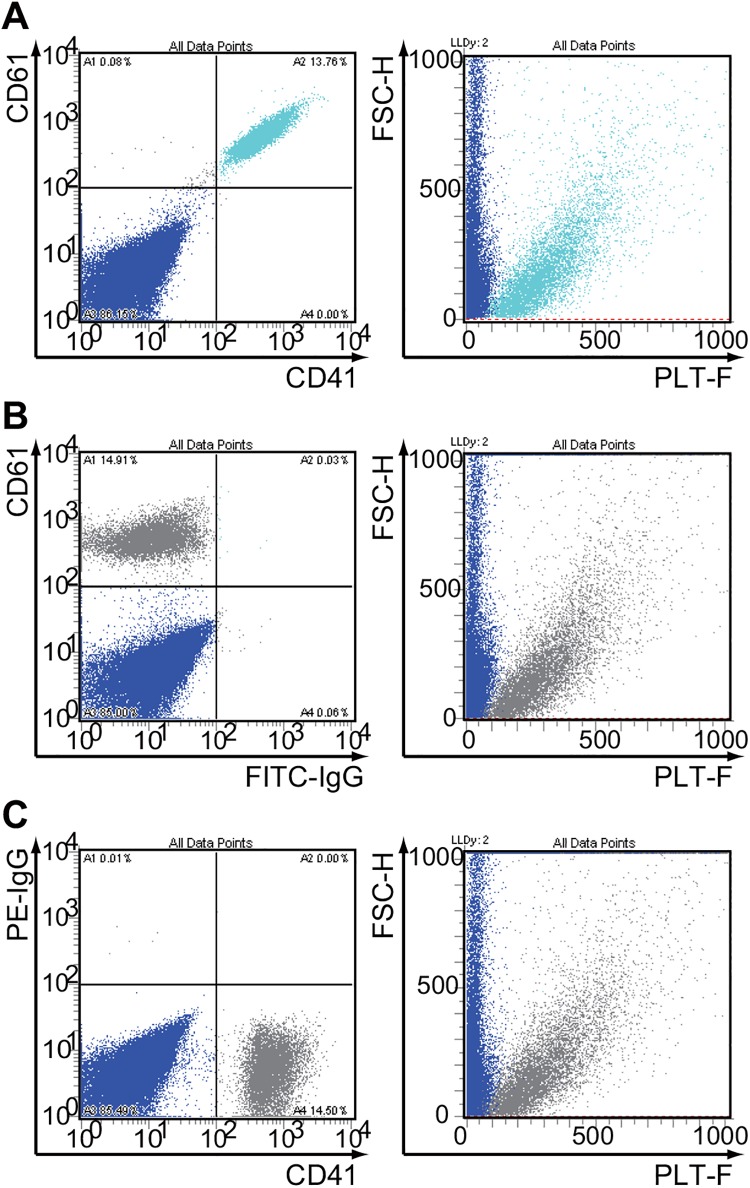
The CD41^+^/CD61^+^ double-positive particles were separated with PLT-F reagents. The mixture of platelets and fragmented erythrocytes that were stained with both FITC-labeled and PE-labeled antibodies were then stained with the PLT-F reagents and examined by flow cytometry. (A) The mixture was stained with both platelet-specific antibodies (anti-CD41 or anti-CD61, left) and PLT-F reagents (middle). (B) FITC-control IgG or (C) PE-control IgG was used as a negative control for fluorescence compensation.

Finally, we regenerated the PLT-F scattergram on a multipurpose flow cytometer and examined the correlation between PLT-F staining and platelet-specific antibody staining (Fig 6). Even though the target of the PLT-F dye is completely different with the CD antigens, this experiment revealed that almost all antibody-labeled platelets could be discriminated from fragmented erythrocytes on the scattergram, which was described in the same manner in the PLT-F system (Fig 5). These results are consistent with previous studies that demonstrated that the PLT-F system was correlated strongly with the immunologic reference method, even when using samples with a platelet count of less than 30 × 10^9^/l [17–20].

## Conclusions

This study suggested that the accurate and precise platelet count of the PLT-F channel is mainly due to the intraplatelet staining property of the PLT-F reagents. It also indicated that the PLT-F channel correctly counts platelets in some abnormal specimens that contain small inhibitory particles, such as fragmented erythrocytes, owing to the properties of the PLT-F reagents.

## Supporting Information

S1 Fig Neither FSC/SSC scattergram nor electrical impedance histogram can separate platelets and fragmented erythrocytes. The same sample as in Fig 4A was developed with side scatter and forward scatter (left) or the diameter of each particle calculated from electrical impedance (EV-Diam, right). Platelets and fragmented erythrocytes could not be separated with these methods.
(EPS)

S1 File Statistical Analysis. **Table A—**p-values between two means of each two platelet counting method. **Table B**—p-values between two means of each two samples.
(DOC)

## References

[1] Kuwana M. Helicobacter. Pylori-associated immune thrombocytopenia: clinical features and pathogenic mechanisms. World J Gastroenterol. 2014;20: 714–723. 10.3748/wjg.v20.i3.714 24574745 PMC3921481

[2] Kashiwagi H, Tomiyama Y. Pathophysiology and management of primary immune thrombocytopenia. Int J Hematol. 2013;98: 24–33. 10.1007/s12185-013-1370-4 23702914

[3] Sola-Visner M. Platelets in the neonatal period: developmental differences in platelet production, function, and hemostasis and the potential impact of therapies. Hematology Am Soc Hematol Educ Program. 2012;2012: 506–511. 10.1182/asheducation-2012.1.506 23233626

[4] Daugirdas JT, Bernardo AA. Hemodialysis effect on platelet count and function and hemodialysis-associated thrombocytopenia. Kidney Int. 2012;82: 147–157. 10.1038/ki.2012.130 22592187

[5] Ortel TL. Heparin-induced thrombocytopenia: when a low platelet count is a mandate for anticoagulation. Hematology Am Soc Hematol Educ Program 225–232 2009. 10.1182/asheducation-2009.1.225 20008202

[6] Rappaport ES, Helbert B, Beissner RS, Trowbridge A. Automated hematology: where we stand. South Med J. 1988;81: 365–370. 3279533 10.1097/00007611-198803000-00018

[7] Dickerhoff R, Von Ruecker A. Enumeration of platelets by multiparameter flow cytometry using platelet-specific antibodies and fluorescent reference particles. Clin Lab Haematol. 1995;17: 163–172. 8536420

[8] Harrison P, Ault KA, Chapman S, Charie L, Davis B, Fujimoto K et al An interlaboratory study of a candidate reference method for platelet counting. Am J Clin Pathol. 2001;115: 448–459. 11242802 10.1309/91PR-E4G6-XBAF-N8DY

[9] Tvedten H. What is your diagnosis? Discrepancy in platelet counts determined using a Sysmex XT-2000 iV hematology analyzer. Erroneous PLT-O due to RBC ghosts. Vet Clin Pathol. 2010;39: 395–396. 10.1111/j.1939-165X.2010.00240.x 20646258

[10] Sandhaus LM, Osei ES, Agrawal NN, Dillman CA, Meyerson HJ. Platelet counting by the coulter LH 750, sysmex XE 2100, and advia 120: a comparative analysis using the RBC/platelet ratio reference method. Am J Clin Pathol. 2002;118: 235–241. 12162684 10.1309/MK3G-MC3V-P06R-PNV2

[11] Hong KH, Kim MJ, Lee KW, Park KU, Kim HS, Song J. Platelet count evaluation using three automated haematology analysers compared with the immunoplatelet reference method, and estimation of possible inadequate platelet transfusion. Int J Lab Hematol. 2009;31: 298–306. 10.1111/j.1751-553X.2008.01032.x 18294237

[12] Trabuio E, Valverde S, Antico F, Manoni F, Gessoni G. Performance of automated platelet quantification using different analysers in comparison with an immunological reference method in thrombocytopenic patients. Blood Transfus. 2009;7: 43–48. 10.2450/2008.0039-08 19290080 PMC2652236

[13] Sehgal K, Badrinath Y, Tembhare P, Subramanian PG, Talole S, Kumar A et al Comparison of platelet counts by CellDyn Sapphire (Abbot Diagnostics), LH750 (Beckman Coulter), ReaPanThrombo immunoplatelet method (ReaMetrix), and the international flow reference method, in thrombocytopenic blood samples. Cytometry B Clin Cytom. 2010;78: 279–285. 10.1002/cyto.b.20515 20229505

[14] Dadu T, Sehgal K, Shaikh A, Khodaiji S. Comparison of platelet counts by sysmex XE 2100 and LH-750 with the international flow reference method in thrombocytopenic patients. Indian J Pathol Microbiol. 2013;56: 114–119. 10.4103/0377-4929.118701 24056646

[15] Cid J, Nascimento JD, Vicent A, Aguinaco R, Escoda L, Ugarriza A et al Evaluation of low platelet counts by optical, impedance, and CD61-immunoplatelet methods: estimation of possible inappropriate platelet transfusion. Transfusion. 2010;50: 795–800. 10.1111/j.1537-2995.2009.02504.x 19951312

[16] Diquattro M, Gagliano F, Calabro GM, Tommasi M, Scott CS, Mancuso G et al Relationships between platelet counts, platelet volumes and reticulated platelets in patients with ITP: evidence for significant platelet count inaccuracies with conventional instrument methods. Int J Lab Hematol. 2009;31: 199–206. 10.1111/j.1751-553X.2007.01025.x 18190589

[17] Briggs C, Longair I, Kumar P, Singh D, Machin SJ. Performance evaluation of the Sysmex haematology XN modular system. J Clin Pathol. 2012;65: 1024–1030. 10.1136/jclinpath-2012-200930 22851510

[18] Schoorl M, Schoorl M, Oomes J, van Pelt J. New fluorescent method (PLT-F) on Sysmex XN2000 hematology analyzer achieved higher accuracy in low platelet counting. Am J Clin Pathol. 2013;140: 495–499. 10.1309/AJCPUAGGB4URL5XO 24045545

[19] Tanaka Y, Tanaka Y, Gondo K, Maruki Y, Kondo T, Asai S et al Performance evaluation of platelet counting by novel fluorescent dye staining in the XN-series automated hematology analyzers. J Clin Lab Anal. 2014;28: 341–348. 10.1002/jcla.21691 24648166 PMC6807536

[20] Seo JY, Lee ST, Kim SH. Performance evaluation of the new hematology analyzer Sysmex XN-series. Int J Lab Hematol. 2015;37: 155–164. 10.1111/ijlh.12254 24815300

[21] Araki J, Jona M, Eto H, Aoi N, Kato H, Suga H et al Optimized preparation method of platelet-concentrated plasma and noncoagulating platelet-derived factor concentrates: maximization of platelet concentration and removal of fibrinogen. Tissue Eng Part C Methods. 2012;18: 176–185. 10.1089/ten.TEC.2011.0308 21951067 PMC3285602

[22] Kanda Y. Investigation of the freely available easy-to-use software 'EZR' for medical statistics. Bone Marrow Transplant. 2013;48: 452–458. 10.1038/bmt.2012.244 23208313 PMC3590441

[23] Wada H, Masuda K, Satoh R, Kakugawa K, Ikawa T, Katsura Y et al Adult T-cell progenitors retain myeloid potential. Nature. 2008;452: 768–772. 10.1038/nature06839 18401412

[24] Harrington LE, Janowski KM, Oliver JR, Zajac AJ, Weaver CT. Memory CD4 T cells emerge from effector T-cell progenitors. Nature. 2008;452: 356–360. 10.1038/nature06672 18322463

[25] Heijnen HF, Debili N, Vainchencker W, Breton-Gorius J, Geuze HJ, Sixma JJ. Multivesicular bodies are an intermediate stage in the formation of platelet alpha-granules. Blood. 1998;91: 2313–2325. 9516129

[26] Flachbartova Z, Kovacech B. Mortalin—a multipotent chaperone regulating cellular processes ranging from viral infection to neurodegeneration. Acta Virol. 2013;57: 3–15. 23530819 10.4149/av_2013_01_3

[27] Michalak M, Groenendyk J, Szabo E, Gold LI, Opas M. Calreticulin, a multi-process calcium-buffering chaperone of the endoplasmic reticulum. Biochem J. 2009;417: 651–666. 10.1042/BJ20081847 19133842

[28] Kono M, Kondo T, Takagi Y, Wada A, Fujimoto K. Morphological definition of CD71 positive reticulocytes by various staining techniques and electron microscopy compared to reticulocytes detected by an automated hematology analyzer. Clin Chim Acta. 2009;404: 105–110. 10.1016/j.cca.2009.03.017 19302987

