# Supplementary material for: Accuracy of a New Platelet Count System (PLT-F) Depends on the Staining Property of Its Reagents
Source: PLoS One. 2015 Oct 23;10(10):e0141311. doi: 10.1371/journal.pone.0141311 (PMC4619826; doi:10.1371/journal.pone.0141311)
Supplement: S1 File — Table A—p-values between two means of each two platelet counting method. Table B—p-values between two means of each two samples. (DOC) [file pone.0141311.s002.doc]

**S1 File. Statistical analysis**

Table A. p-values between two means of each two platelet counting method

|  | Donor [23] | | Donor [24] | |
| --- | --- | --- | --- | --- |
|  | Control | fRBC | Control | fRBC |
| PLTF vs. PLTO | 0.78 | 2.00E-16 | 0.86 | 2.00E-16 |
| PLTF vs. PLTI | 4.40E-08 | 2.00E-16 | 1.70E-05 | 2.00E-16 |
| PLTO vs. PLTI | 7.70E-07 | 4.50E-07 | 9.90E-07 | 7.20E-13 |

Welch’s t-test was used for pairwise comparison. The Bonferroni method was used for p-value adjustment. A p-value equal to or below 0.05 was considered statistically significant.

Table B. p-values between two means of each two samples

|  | Control vs. fRBC | |
| --- | --- | --- |
|  | Donor [23] | Donor [24] |
| PLT-F | 3.81E-12 | 2.27E-14 |
| PLT-O | 2.48E-15 | 1.11E-16 |
| PLT-I | 3.03E-28 | 1.07E-32 |

Welch’s t-test was used for a pairwise comparison. The Bonferroni method was used for p-value adjustment. A p-value equal to or below 0.05 was considered statistically significant.
